# Managing capivasertib-induced hyperglycemia: a report of 3 cases

**DOI:** 10.1210/jcemcr/luag273

**Published:** 2026-09-16

**Authors:** Katherine Cuan, Sun Young Oh, Jovan Milosavljevic, Beatrice Y Wong

**Affiliations:** Division of Endocrinology, Department of Medicine, Montefiore Medical Center, Albert Einstein College of Medicine, Bronx, NY 10467, USA; Division of Hematology and Oncology, Department of Medicine, Montefiore Medical Center, Albert Einstein College of Medicine, Bronx, NY 10467, USA; Division of Endocrinology, Department of Medicine, Montefiore Medical Center, Albert Einstein College of Medicine, Bronx, NY 10467, USA; Division of Endocrinology, Department of Medicine, Montefiore Medical Center, Albert Einstein College of Medicine, Bronx, NY 10467, USA

**Keywords:** insulin resistance, capivasertib, AKT inhibitor

## Abstract

Phosphoinositide 3-kinase/protein kinase B (AKT) pathway inhibition is an important therapeutic strategy for hormone receptor positive and human epidermal growth factor receptor 2 negative advanced breast cancer. Capivasertib, an oral pan-AKT inhibitor, improves progression-free survival in combination with fulvestrant but is frequently associated with hyperglycemia. Despite this recognized adverse effect, consensus management guidelines are lacking. We describe 3 patients who developed capivasertib-associated hyperglycemia, 2 of whom had severe hyperglycemia with alpelisib therapy, a phosphoinositide 3-kinase inhibitor. Hyperglycemia developed early and was marked by postprandial excursions. All patients were treated with insulin-sensitizing therapies. Preemptive initiation or intensification of antihyperglycemic therapy before capivasertib exposure attenuated hyperglycemia severity. These cases highlight the insulin-resistant phenotype of AKT inhibitor-associated hyperglycemia and support early metabolic assessment, postprandial glucose monitoring, and preferential use of insulin-sensitizing therapies to minimize treatment interruption.

## Introduction

Advanced breast cancer therapy increasingly targets the phosphoinositide 3-kinase/protein kinase B (PI3K/AKT) signaling pathway, which regulates cellular growth, proliferation, and survival [1]. Approved PI3K/AKT inhibitors for hormone receptor positive (HR+), human epidermal growth factor receptor 2 negative (HER2–) advanced breast cancer include the PI3Kα inhibitors alpelisib and inavolisib, and the AKT inhibitor capivasertib [1]. On a standard treatment cycle, capivasertib is administered at 400 mg twice daily on a 4 days on/3 days off schedule, in combination with fulvestrant [2].

Hyperglycemia is a common adverse effect of PI3K/AKT inhibitors and may limit therapeutic efficacy through treatment interruptions, dose reductions, or discontinuation [3]. In CAPItello-291, all-grade hyperglycemia occurred in 16.3% of patients receiving capivasertib, with grade ≥3 events in 2.3% [4]. The PI3K/AKT inhibition disrupts insulin signaling, causing impaired glucose uptake, hepatic gluconeogenesis, and insulin resistance, supporting the use of insulin-sensitizing or insulin-independent therapies, such as metformin and sodium-glucose cotransporter 2 inhibitors (SGLT2i), for PI3K/AKT-associated hyperglycemia [3, 5, 6]. Despite established metabolic adverse effects, consensus guidelines for evaluating and managing capivasertib-associated hyperglycemia are lacking.

As PI3K/AKT inhibitor use expands, recognizing and managing treatment-associated hyperglycemia are important to optimize oncologic outcomes. We describe 3 patients with capivasertib-associated hyperglycemia, 2 with prior alpelisib-associated hyperglycemia and discuss management strategies.

## Case presentation

### Case 1

An 86-year-old woman with prediabetes, body mass index (BMI) of 24 kg/m^2^ (reference range, 18.5-24.9 kg/m^2^), hypertension (HTN), and metastatic HR+/HER2- breast cancer presented with hyperglycemia following capivasertib initiation at 400 mg twice daily 1 month prior to presentation. In the year preceding capivasertib therapy, random serum glucose values ranged from 98 (International System of Units [SI]: 5.4 mmol/L) to 187 mg/dL (SI: 10.4 mmol/L; reference range, 70-140 mg/dL [SI: 3.9-7.8 mmol/L]). Although she had a history of prediabetes, prior hemoglobin A1c (HbA1c) measurements were not available.

### Case 2

A 71-year-old woman with Class I obesity (BMI 30 kg/m^2^), type 2 diabetes (T2D), HTN, hyperlipidemia (HLD), metabolic dysfunction-associated steatotic liver disease, and metastatic HR+/HER2- breast cancer was previously treated with alpelisib, which was discontinued due to nonadherence. She was transitioned to capivasertib due to disease progression. Prior to alpelisib, she was not receiving antihyperglycemic medications, with a baseline HbA1c of 7.3% (SI: 56 mmol/mol; reference range, <5.7% [SI: <39 mmol/mol]).

Following alpelisib initiation, she developed grade 3 hyperglycemia and HbA1c peaked at 9.1% (SI: 76 mmol/mol). Metformin 1000 mg twice daily and pioglitazone 15 mg daily were initiated. Alpelisib was held, resumed at a reduced dose, then discontinued due to intermittent adherence with HbA1c improving to 6.0% (SI: 42 mmol/mol). One month later, pioglitazone was preemptively increased to 30 mg daily, and metformin was continued at the same dose for capivasertib initiation 5 days later.

### Case 3

A 79-year-old woman with hypothyroidism, T2D, HTN, HLD, BMI 24 kg/m^2^, and metastatic HR+/HER2- breast cancer previously developed hyperglycemia with alpelisib. Prior to alpelisib initiation, her HbA1c was 6.5% (SI: 48 mmol/mol) on sitagliptin 25 mg daily.

Following alpelisib initiation, she developed grade 4 hyperglycemia with serum glucose peaking at 642 mg/dL (SI: 35.6 mmol/L) and HbA1c increasing to 9.3% (SI: 78 mmol/mol). Metformin 500 mg twice daily and pioglitazone 15 mg daily were started without improvement. Alpelisib was ultimately discontinued due to hyperglycemia.

Over the following months, she tried dulaglutide, linagliptin, and repaglinide. She self-discontinued metformin during this period. Glucose levels transiently improved with HbA1c decreasing to 6.8% (SI: 51 mmol/mol). A year later, HbA1c rose to 9% (SI: 75 mmol/mol) while taking linagliptin 5 mg daily, repaglinide 1 mg 3 times daily, and pioglitazone 30 mg daily. One month later, she was started on capivasertib.

## Diagnostic assessment

### Case 1

Two days after capivasertib initiation, postprandial serum glucose was 269 mg/dL (SI: 14.9 mmol/L) and HbA1c was 6.1% (SI: 43 mmol/mol). Capivasertib was held on Day 16 due to polyuria and severe hyperglycemia, with postprandial serum glucose of 492 mg/dL (SI: 27.3 mmol/L). While off capivasertib, c-peptide was 2.42 ng/mL (SI: 0.81 nmol/L; reference range, 1.0-4.0 ng/mL [SI: 0.33-1.33 nmol/L]), serum insulin was 4.1 μU/mL (SI: 24.6 pmol/L; reference range, <20 μU/mL [SI: 120 pmol/L]), and a concurrent random serum glucose of 105 mg/dL (SI: 5.82 mmol/L).

### Case 2

While on capivasertib, HbA1c was 6.2% (SI: 44 mmol/mol), c-peptide was 2.43 ng/mL (SI: 0.77 nmol/L), serum insulin was 5.7 μU/mL (SI: 34.2 pmol/L), and a concurrent fasting serum glucose of 82 mg/dL (SI: 4.55 mmol/L).

### Case 3

Ten days after capivasertib initiation, the dose was reduced to 320 mg twice daily due to gastrointestinal toxicity and capillary glucose readings up to 424 mg/dL (SI: 23.5 mmol/L). Diarrhea resolved with dose reduction; however, capillary glucose values remained >400 mg/dL (SI: >22.2 mmol/L). Capivasertib was held for 1 week and resumed at the reduced dose after fasting glucose improved. While on capivasertib, her c-peptide was 2.79 ng/mL (SI: 0.92 nmol/L), serum insulin was 6.2 μU/mL (SI: 37.2 pmol/L), and a simultaneous fasting serum glucose was 165 mg/dL (SI: 9.16 mmol/L).

## Treatment

### Case 1

**Figure 1:**
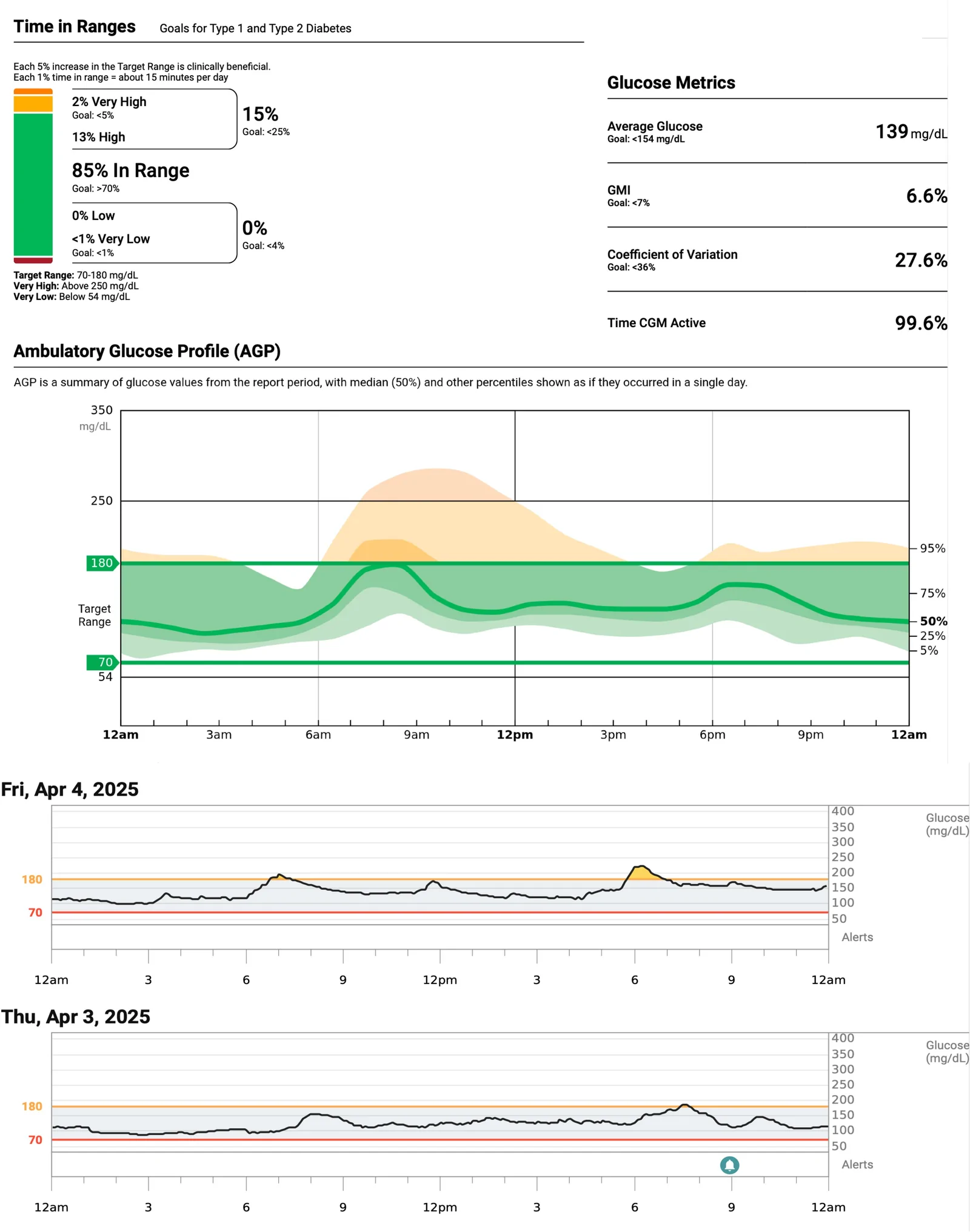
Case 1—continuous glucose monitoring (CGM) tracing following initiation of metformin/pioglitazone (850 mg/15 mg daily) and resumption of capivasertib at 400 mg twice daily. CGM demonstrated 85% time in range (TIR; glucose 70-180 mg/dL; SI: 3.9-10 mmol/L), mean glucose of 139 mg/dL (SI: 7.7 mmol/L), and a peak postprandial glucose of 290 mg/dL (SI: 16.1 mmol/L).

She was started on combination metformin/pioglitazone 850 mg/15 mg daily, and capivasertib was resumed 2 days later at the standard dose. A 2 week continuous glucose monitoring (CGM) trial showed 85% time in range (TIR; glucose 70-180 mg/dL; SI: 3.9-10 mmol/L), an average glucose of 139 mg/dL (SI: 7.7 mmol/L), and a peak postprandial glucose of 290 mg/dL (SI: 16.1 mmol/L; Fig. 1).

The patient was later hospitalized for profuse vomiting and poor oral intake. On presentation, she had an anion gap metabolic acidosis, acute kidney injury, HbA1c of 6.8% (SI: 51 mmol/mol), serum glucose of 205 mg/dL (SI: 11.4 mmol/L), and negative urine ketones. Serum ketones were not measured. She improved with intravenous fluids and bicarbonate infusion. Capivasertib and metformin/pioglitazone were held on admission and discontinued on discharge.

### Case 2

**Figure 2:**
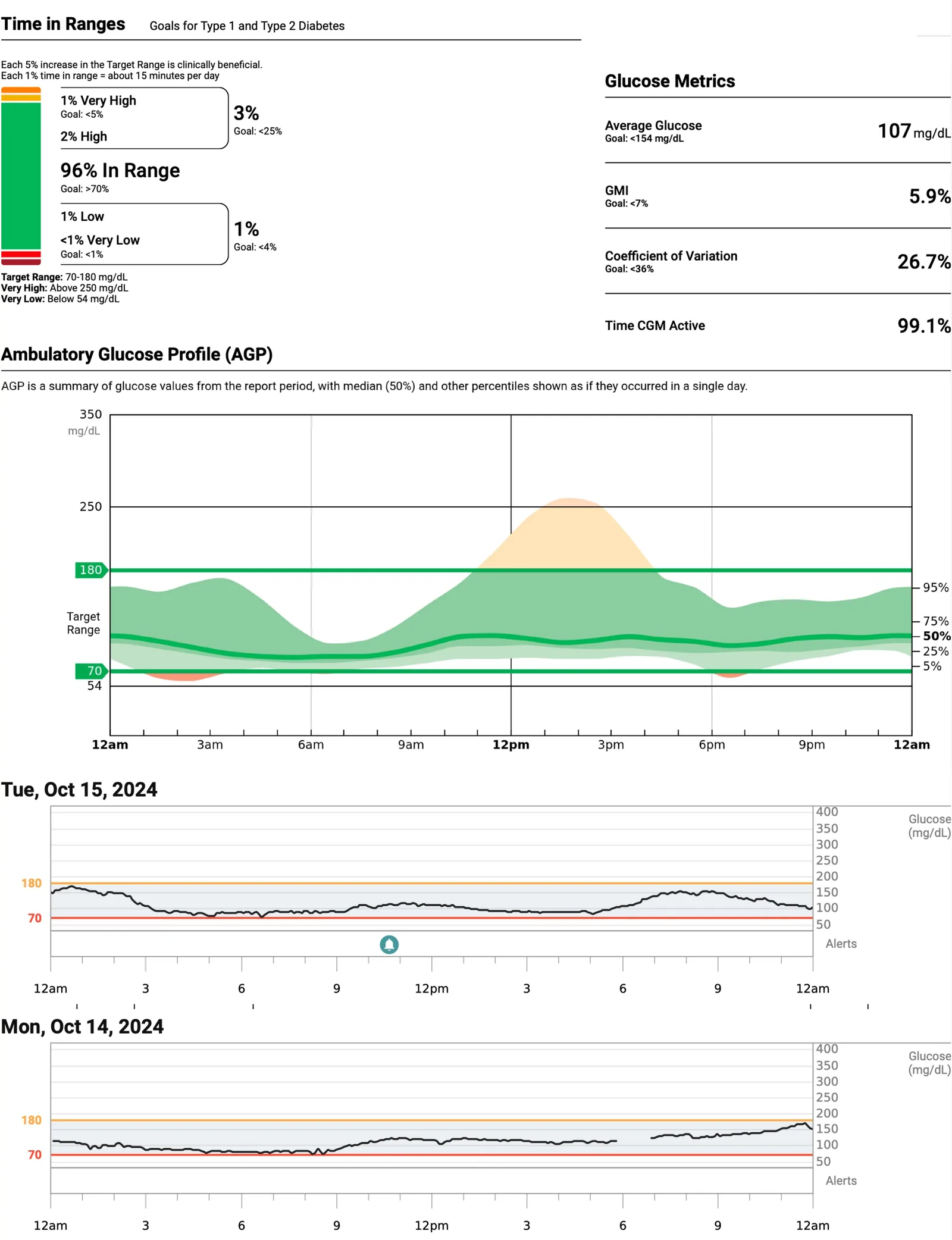
Case 2—continuous glucose monitoring (CGM) tracing following preemptive pioglitazone dose escalation to 30 mg daily, with metformin continued at the same dose, prior to capivasertib initiation. CGM demonstrated 96% time in range (TIR; glucose 70-180 mg/dL) (SI: 3.9-10 mmol/L), mean glucose of 107 mg/dL (SI: 5.9 mmol/L), and a single postprandial excursion to 271 mg/dL (SI: 15.0 mmol/L).

A 2 week CGM trial showed 96% TIR, an average glucose of 107 mg/dL (SI: 5.9 mmol/L) and a single postprandial excursion to 271 mg/dL (SI: 15.0 mmol/L; Fig. 2). Due to neutropenia, capivasertib was reduced to 320 mg twice daily 2 weeks later.

### Case 3

**Figure 3:**
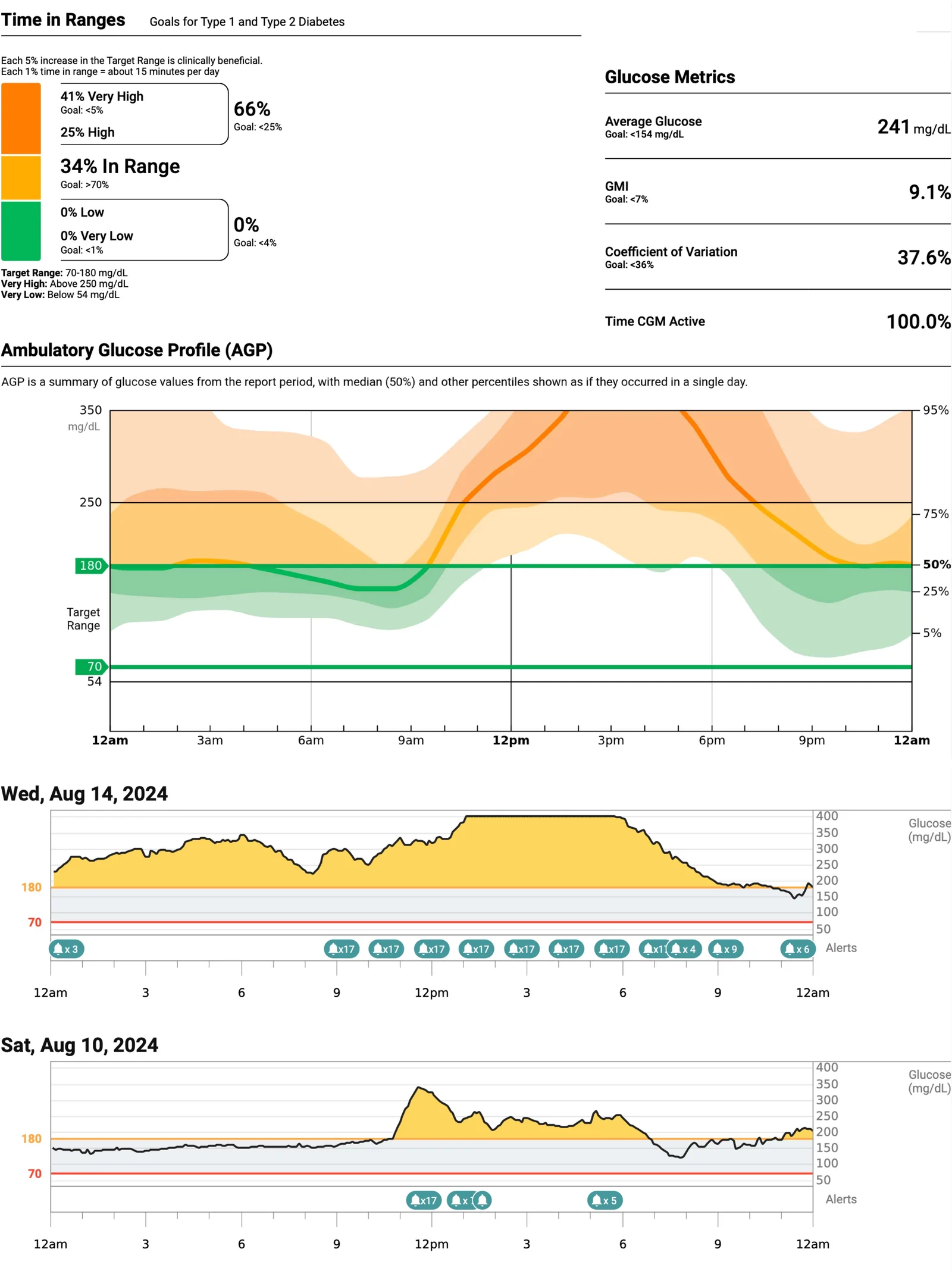
Case 3—continuous glucose monitoring (CGM) tracing following capivasertib initiation. CGM demonstrated 34% time in range (TIR; glucose 70-180 mg/dL; SI: 3.9-10 mmol/L), mean glucose of 241 mg/dL (SI: 13.4 mmol/L), and marked postprandial hyperglycemia that was more pronounced on treatment days than nontreatment days.

Two weeks after capivasertib resumption, postprandial capillary glucose again increased to 395 mg/dL (SI: 16.4 mmol/L). HbA1c peaked at 9.6% (SI: 81 mmol/mol). Empagliflozin 10 mg daily was added to her regimen. A 2-week CGM trial showed 34% TIR with an average glucose of 241 mg/dL (SI: 13.4 mmol/L) and more pronounced postprandial hyperglycemia on treatment days (Fig. 3). Empagliflozin was increased to 25 mg daily.

## Outcome and follow-up

### Case 1

A month later, capivasertib was restarted at 320 mg twice daily but was permanently discontinued after she experienced recurrent intractable vomiting attributed to gastrointestinal toxicity. Metformin/pioglitazone was not resumed. Two months later, HbA1c was 5.8% (SI: 40 mmol/mol). Nine months later, concurrent labs showed c-peptide was 3.2 ng/mL (SI: 1.06 nmol/L), serum insulin was 7.2 μU/mL (SI: 43.2 pmol/L), and random serum glucose was 115 mg/dL (SI: 6.4 mmol/L).

### Case 2

Two weeks later, capivasertib was discontinued due to an HR status change. She continued metformin 1000 mg twice daily and pioglitazone 30 mg daily. Seven months later, she was hospitalized for sepsis with adequate glucose control off all antihyperglycemic agents. Pioglitazone was discontinued on discharge. Within weeks of discharge, the patient transitioned to hospice care and subsequently died.

### Case 3

Two months later, capivasertib was discontinued due to disease progression, and empagliflozin was reduced to 10 mg daily in anticipation of glycemic improvement. She was started on an alternative oncologic agent. Eight months later, repeat HbA1c was 6.3% (SI: 45 mmol/mol), and repaglinide was discontinued.

## Discussion

The PI3K/AKT pathway alterations occur in ∼40% of breast cancers and contribute to oncogenesis [1, 7]. Capivasertib improves progression-free and overall survival in HR+/HER2- advanced breast cancer when combined with fulvestrant [4]. Because this pathway is central to glucose metabolism, hyperglycemia is a common adverse effect, reported in 16.9% of patients receiving capivasertib plus fulvestrant compared with 4.0% in the placebo group [2]. Identified risk factors include elevated baseline BMI and preexisting dysglycemia [2, 3]. Protein kinase B activation mediates insulin-driven glucose uptake, glycogen synthesis, and glycolysis. Thus, pharmacologic inhibition of this pathway results in insulin resistance and hyperglycemia [7, 8]. The severity of insulin resistance can be profound, as evidenced by reports of prolonged high-dose insulin infusions for capivasertib-associated diabetic ketoacidosis [9].

Despite these observations, guidance for capivasertib-associated hyperglycemia in real-world practice remains limited. In CAPItello-291, few patients had pretreatment diabetes (9.6%) and most had well-controlled baseline glycemia (HbA1c <6.5% [SI: 48 mmol/mmol] in 93.5%), leaving uncertainty for patients treated outside clinical trials [2]. The insulin-resistant state induced by AKT inhibition supports preferential use of insulin-sensitizing or insulin-independent therapies, although no consensus guidelines exist specifically for capivasertib. Recommendations for alpelisib support prophylactic metformin in higher-risk patients, an approach that may be extrapolated to capivasertib [10]. Insulin, SGLT2i, and sulfonylureas have been used less frequently. Available data suggest SGLT2i may be associated with more favorable outcomes, although Case 3 suggests they may be insufficient when baseline glycemia is suboptimal [3, 5]. Preclinical models suggest possible reactivation of the PI3K/AKT pathway with high insulin levels [11]. Thus, therapies that increase circulating insulin levels, such as insulin or sulfonylureas, may theoretically counteract the antitumor effects by reactivating downstream signaling [3].

Our cases highlight shared metabolic consequences of PI3K/AKT inhibition, 2 with prior alpelisib-associated hyperglycemia. All patients received pioglitazone and metformin, an approach aligned with the insulin-resistant mechanism and avoiding direct stimulation of insulin secretion. In Case 1, marked hyperglycemia developed within 2 days of capivasertib initiation, earlier than the median onset of 15 days reported in CAPItello-291 [2]. Following preemptive initiation of metformin and pioglitazone, hyperglycemia severity was attenuated after capivasertib resumption. A similar pattern was observed in Case 2. In contrast, Case 3 underscores the importance of optimizing glucose levels prior to capivasertib initiation, as suboptimal baseline status was associated with more severe and persistent hyperglycemia (Table 1). Preserved c-peptide across all 3 cases is suggestive of insulin resistance rather than impaired secretion. Glycemic normalization following capivasertib discontinuation in Cases 1 and 3, without antihyperglycemic therapy in Case 1, suggests the insulin resistance is pharmacodynamically driven and largely reversible. These observations highlight the value of baseline HbA1c and pretreatment glucose monitoring with CGM, corroborating prior reports demonstrating its utility in capivasertib-associated hyperglycemia [12-14]. To our knowledge, this is the first case series to demonstrate successful use of metformin and pioglitazone for capivasertib-associated hyperglycemia, as prior reports have relied on insulin for severe presentations or found metformin ineffective [9, 12, 15]. Meaningful glycemic outcomes were achieved despite variability in treatment duration, even in patients with preexisting diabetes or prior severe hyperglycemia on alpelisib.

**Table 1:** Clinical summary of 3 patients with capivasertib-associated hyperglycemia

	Case 1	Case 2	Case 3
**Baseline characteristics**			
Age	86 years	71 years	79 years
BMI	24 kg/m^2^	30 kg/m^2^	24 kg/m^2^
Baseline glycemic status	Prediabetes HbA1c unavailable Random glucose 98-187 mg/dL (SI: 5.4-10.4 mmol/L)	T2D, unmedicated HbA1c 7.3% (SI: 56 mmol/mol)	T2D on sitagliptin 25 mg daily HbA1c 6.5% (SI: 48 mmol/mol)
Comorbidities	HTN	HTN, HLD, MASLD	HTN, HLD, hypothyroidism
**Prior alpelisib exposure**			
Prior alpelisib use/reason stopped	None	Yes/nonadherence	Yes/hyperglycemia
Peak HbA1c on alpelisib	N/A	9.1% (SI: 76 mmol/mol)	9.3% (SI: 78 mmol/mol) Glucose 642 mg/dL (SI: 35.6 mmol/L)
HbA1c and medications at capivasertib initiation	HbA1c unavailable No antihyperglycemic medications	HbA1c 6.0% (SI: 42 mmol/mol) Metformin 1000 mg BID + pioglitazone 30 mg daily	HbA1c 9.0% (SI: 75 mmol/mol) Linagliptin 5 mg daily + repaglinide 1 mg TID + pioglitazone 30 mg daily
**Capivasertib course and hyperglycemia**			
Starting dose/adjustments	400 mg BID → reduced to 320 mg BID Permanently discontinued (GI toxicity)	400 mg BID → reduced to 320 mg BID (neutropenia) Discontinued (HR status change)	400 mg BID → reduced to 320 mg BID (GI toxicity) Held × 1 week; resumed; discontinued (disease progression)
Onset/peak glucose on capivasertib	Day 2: 269 mg/dL (SI: 14.9 mmol/L) Day 16 (held): 492 mg/dL (SI: 27.3 mmol/L)	Single excursion to 271 mg/dL (SI: 15.0 mmol/L)	Day 10: up to 424 mg/dL (SI: 23.5 mmol/L) Peak: >400 mg/dL (SI: >22.2 mmol/L)
Peak HbA1c on capivasertib	6.8% (SI: 51 mmol/mol)	6.2% (SI: 44 mmol/mol)	9.6% (SI: 81 mmol/mol)
Antihyperglycemic treatment added	Metformin/pioglitazone 850/15 mg daily	Preexisting metformin 1000 mg BID + pioglitazone 30 mg daily (no changes needed)	Empagliflozin added 10 mg → 25 mg daily (added to pre-existing regimen)
CGM (2 week trial) findings	TIR 85% Mean glucose 139 mg/dL (SI: 7.7 mmol/L)	TIR 96% Mean glucose 107 mg/dL (SI: 5.9 mmol/L)	TIR 34% Mean glucose 241 mg/dL (SI: 13.4 mmol/L) Postprandial spikes worse on treatment days
**Insulin-resistance markers (c-peptide, insulin, and glucose)**			
Timing of labs	Off capivasertib (held day 16)	On capivasertib	On capivasertib
C-peptide	2.42 ng/mL (SI: 0.81 nmol/L)	2.43 ng/mL (SI: 0.77 nmol/L)	2.79 ng/mL (SI: 0.92 nmol/L)
Serum insulin	4.1 μU/mL (SI: 24.6 pmol/L)	5.7 μU/mL (SI: 34.2 pmol/L)	6.2 μU/mL (SI: 37.2 pmol/L)
Concurrent serum glucose	105 mg/dL (SI: 5.82 mmol/L), random	82 mg/dL (SI: 4.55 mmol/L), fasting	165 mg/dL (SI: 9.16 mmol/L), fasting
Postdiscontinuation labs	C-peptide 3.2 ng/mL (SI: 1.06 nmol/L) Insulin 7.2 μU/mL (SI: 43.2 pmol/L) Random glucose 115 mg/dL (SI: 6.4 mmol/L) HbA1c 5.8% (SI: 40 mmol/mol)	Not available	HbA1c 6.3% (SI: 45 mmol/mol)
**Glycemic outcome after capivasertib discontinuation**			
HbA1c after discontinuation	5.8% (SI: 40 mmol/mol) at 2 months	Not available Glucose adequate off all antihyperglycemic agents during hospitalization	6.3% (SI: 45 mmol/mol) at 8 months
Medication changes after discontinuation	Metformin/pioglitazone not resumed	Pioglitazone discontinued; patient transitioned to hospice	Empagliflozin reduced to 10 mg daily Repaglinide discontinued

Abbreviations: BID, twice daily; BMI, body mass index; CGM, continuous glucose monitor; GI, gastrointestinal; HbA1c, hemoglobin A1c; HLD, hyperlipidemia; HR, hormone receptor; HTN, hypertension; MASLD, metabolic dysfunction-associated steatotic liver disease; T2D, type 2 diabetes; TID, 3 times daily; TIR, time in range (glucose 70-180 mg/dL [3.9-10.0 mmol/L]).

These cases underscore the importance of endocrinology–oncology collaboration and early postprandial glucose monitoring. Further studies are needed to define optimal prevention and management of PI3K/AKT inhibitor-associated hyperglycemia while preserving oncologic efficacy.

## Learning points

- Capivasertib-associated hyperglycemia is driven by AKT inhibition-induced insulin resistance and can develop within days of initiation.
- Preemptive or early intensification of antihyperglycemic therapy may mitigate early hyperglycemia severity and prevent treatment interruptions.
- Insulin-sensitizing therapies, particularly metformin and pioglitazone, target the underlying mechanism of capivasertib-associated hyperglycemia and can enable continued treatment.
- Baseline metabolic assessment, early postprandial glucose monitoring, and endocrinology–oncology collaboration are essential to balance glycemic control with oncologic efficacy.

## Contributors

All authors made individual contributions to authorship. B.Y.W. and S.Y.O. were involved in the diagnosis and management of the patients. K.C., J.M., and B.Y.W. were involved in the overall design of this report and contributed to acquisition and organization of clinical data. All authors reviewed and approved the final draft.

## Data Availability

Data sharing is not applicable to this article, as no datasets were generated or analyzed during the current study.

## References

[1] Andrikopoulou A, Chatzinikolaou S, Panourgias E, et al The emerging role of capivasertib in breast cancer. Breast. 2022;63:157‐167. 35398754 10.1016/j.breast.2022.03.018 PMC9011110

[2] Rugo HS, Oliveira M, Howell SJ, et al Capivasertib and fulvestrant for patients with hormone receptor-positive advanced breast cancer: characterization, time course, and management of frequent adverse events from the phase III CAPItello-291 study. ESMO Open. 2024;9(9):103697. 39241495 10.1016/j.esmoop.2024.103697 PMC11406080

[3] Liu D, Weintraub MA, Garcia C, et al Characterization, management, and risk factors of hyperglycemia during PI3K or AKT inhibitor treatment. Cancer Med. 2022;11(8):1796‐1804. 35212193 10.1002/cam4.4579 PMC9041081

[4] Turner NC, Oliveira M, Howell SJ, et al Capivasertib in hormone receptor-positive advanced breast cancer. N Engl J Med. 2023;388(22):2058‐2070. 37256976 10.1056/NEJMoa2214131 PMC11335038

[5] Blow T, Hyde PN, Falcone JN, et al Treating alpelisib-induced hyperglycemia with very low carbohydrate diets and sodium-glucose co-transporter 2 inhibitors: a case series. Integr Cancer Ther. 2021;20:15347354211032283. 34259084 10.1177/15347354211032283 PMC8283040

[6] Shen S, Chen Y, Carpio A, Chang C, Iyengar NM. Incidence, risk factors, and management of alpelisib-associated hyperglycemia in metastatic breast cancer. Cancer. 2023;129(24):3854‐3861. 37743730 10.1002/cncr.34928 PMC10863751

[7] Nitulescu GM, Van De Venter M, Nitulescu G, et al The Akt pathway in oncology therapy and beyond (review). Int J Oncol. 2018;53(6):2319‐2331. 30334567 10.3892/ijo.2018.4597 PMC6203150

[8] Li X, Hu S, Cai Y, Liu X, Luo J, Wu T. Revving the engine: PKB/AKT as a key regulator of cellular glucose metabolism. Front Physiol. 2023;14:1320964. 38264327 10.3389/fphys.2023.1320964 PMC10804622

[9] Nicolich-Henkin S, Waters L, Bansal N, Klek S. Glycemic derangements with capivasertib—from hyperglycemia to diabetic ketoacidosis: a report of 3 cases. JCEM Case Rep. 2025;3(10):luaf198. 40895496 10.1210/jcemcr/luaf198 PMC12395548

[10] Gallagher EJ, Moore H, Lacouture ME, et al Managing hyperglycemia and rash associated with alpelisib: expert consensus recommendations using the Delphi technique. NPJ Breast Cancer. 2024;10(1):12. 38297009 10.1038/s41523-024-00613-x PMC10831089

[11] Hopkins BD, Pauli C, Du X, et al Suppression of insulin feedback enhances the efficacy of PI3K inhibitors. Nature. 2018;560(7719):499‐503. 30051890 10.1038/s41586-018-0343-4 PMC6197057

[12] Takahashi H, Hatayama S, Izumi T. Characteristic glycemic patterns during capivasertib treatment identified by continuous glucose monitoring: a case report. Intern Med. 2025;65(14):1937‐1942. 41407340 10.2169/internalmedicine.6353-25 PMC13447531

[13] Sasada S, Ohno H, Taonouchi H, et al Dynamic changes of blood glucose level during capivasertib treatment in metastatic breast cancer: a case report. Diabetol Int. 2025;16(4):851‐854. 41111584 10.1007/s13340-025-00841-x PMC12532970

[14] Suzuki Y, Yamaguchi T, Uchida T, et al Continuous glucose monitoring reveals bimodal glycemic patterns during and outside capivasertib administration. J Diabetes Investig. 2026;17(4):698‐701. 10.1111/jdi.70261 PMC13042936 41685403

[15] Nomoto Y, Mukai M, Eguchi Y, Yoshinaka H, Shinden Y, Nakajo A. A case of grade 4 hyperglycemia induced by capivasertib and successfully managed with early insulin intervention. Surg Case Rep. 2025;11(1):25-0514. 10.70352/scrj.cr.25-0514 PMC12702576 41399756

